# Sickle cell disease and H3Africa: enhancing genomic research on cardiovascular diseases in African patients

**DOI:** 10.5830/CVJA-2015-040

**Published:** 2015

**Authors:** Ambroise Wonkam, Julie Makani, Solomon Ofori-Aquah, Obiageli E Nnodu, Marsha Treadwell, Charmaine Royal, Kwaku Ohene-Frempong

**Affiliations:** Division of Human Genetics, Faculty of Health Sciences, University of Cape Town, South Africa; Muhimbili University of Health and Allied Sciences, Dar-Es-Salaam, Tanzania; Center for Translational and International Hematology, University of Pittsburgh, Pittsburgh, USA; Department of Haematology and Blood Transfusion, College of Health Sciences, University of Abuja, Abuja, Nigeria/Department of Haematology and Blood Transfusion, University of Abuja Teaching Hospital, Gwagwalada, Abuja, Nigeria; Hematology/Oncology Department, UCSF Benioff Children’s Hospital, Oakland, USA; Department of African and African American Studies, Duke University, Durham, USA; Children’s Hospital of Philadelphia, Comprehensive Sickle Cell Centre, Philadelphia, USA

**Keywords:** sickle cell disease, stroke, kidney diseases, pulmonary hypertension, genetics, Africa

## Abstract

**Background:**

Sickle cell disease (SCD) has a high prevalence in sub-Saharan Africa. There are several cardiovascular phenotypes in SCD that contribute to its morbidity and mortality.

**Discussion:**

SCD is characterised by marked clinical variability, with genetic factors playing key modulating roles. Studies in Tanzania and Cameroon have reported that singlenucleotide polymorphisms in *BCL11A* and *HBS1L-MYB* loci and co-inheritance of alpha-thalassaemia impact on foetal haemoglobin levels and clinical severity. The prevalence of overt stroke among SCD patients in Cameroon (6.7%) and Nigeria (8.7%) suggests a higher burden than in high-income countries. There is also some evidence of high burden of kidney disease and pulmonary hypertension in SCD; however, the burden and genetics of these cardiovascular conditions have seldom been investigated in Africa.

**Conclusions:**

Several H3Africa projects are focused on cardiovascular diseases and present major opportunities to build genome-based research on existing SCD platforms in Africa to transform the health outcomes of patients.

## Abstract

Sickle cell disease (SCD) is a genetic disorder of public health significance with high prevalence, high mortality rate and limited interventions. An estimated 305 800 births are affected annually worldwide by homozygous SCD (SCD-SS), nearly two-thirds of this incidence occurs in Africa.^1^ This estimate does not include SCD-SC, which is more prevalent than SCD-SS in some West African countries.

Although the first clinical description of SCD occurred over 100 years ago and this condition was described in 1949 as the first molecular disease, to date only one drug, hydroxyurea, is available for its specific treatment.^2^ Furthermore, despite the evidence from high-income countries that new-born screening (NBS) and comprehensive care are associated with a 70% reduction in early childhood deaths,^3^ and can have a significant impact on reducing morbidity,^4^,^5^ few African countries have programmes dedicated to NBS, follow-up care, family and patient education and counselling, and prevention and treatment of disease complications. As a consequence, in sub-Saharan Africa, mortality rates are high before the age of five years and estimates suggest that without intervention, up to 90% of affected infants may die in childhood.^6^,^7^

## The role of genomic research to improve health of SCD patients: preliminary data from Cameroon and Tanzania

## Genomics of foetal haemoglobin-promoting loci

Advancement in genomic research offers an unprecedented opportunity to address the health challenges of SCD in an integrated manner. As a Mendelian disorder caused by a single gene mutation on the β-globin gene (β^Glu6Val^) on chromosome 11, there is considerable phenotypic diversity in SCD, due largely to the influence of genetic and environmental factors.^8^-^10^

Although there are several key phenotypes (anaemia, stroke, infections), foetal haemoglobin (HbF) has emerged as a central disease modifier; importantly, the expression of this modifier is amenable to therapeutic manipulation.^11^,^12^ Genetic variants at three principal loci, *BCL11A, HBS1L-MYB* and the HBB cluster account for 10–20% of HbF variation among SCD patients in the USA, Brazil and the UK.^8^,^9^

Initial studies in Tanzania^13^ and recently in Cameroon^14^,^15^ have shown that single-nucleotide polymorphisms (SNPs) in the *BCL11A* loci are prevalent in both Tanzanian and Cameroonian patients [minor allele frequency (MAF) of rs4671393 = 0.30], with significant association of these SNPs with HbF (Table 1). These studies have also shown that rs9399137, which acts as a tagging SNP for the *HMIP-2* sub-locus in European populations,^10^ occurred at a low frequency in both Cameroonian^14^ and Tanzanian patients.^13^ Nevertheless, in the *HMIP-2* sub-locus, there was a much higher MAF of rs9389269 in Cameroonian (0.18)^14^ compared to the Tanzanian SCD patients (0.03).^13^ This observation could indicate a high degree of variation in the MAF of this SNP among SCD patients in African population groups.^16^

**Table 1:** Foetal haemoglobin association results for SNPs at the *BCL11A, HBS1L-MYB* and beta-globin loci in the Cameroonian and Tanzanian sickle cell anaemia cohort

*Locus*	*Genomic variations*			*HbSS Cameroon (n = 596)14*			*HbSS Tanzania (n = 1 124)13*		
	*SNP*	Position on the chromosome*	*Allele change*	*MAF*	*Effect size*	*p-value*	*MAF*	*Effect size*	*p-value*
Chromosome 2									
*BCL11A*	rs11886868	60720246	T>C	0.31	0.167	0.0129	0.26	–0.406	3.00E-30
*BCL11A*	rs4671393	60720951	G>A	0.3	0.201	0.0062	0.3	–0.412	3.90E-28
Chromosome 6									
*HBS1L-MYB*	rs28384513	135376209	A>C	0.2	–0.3002	0.0002	0.21	–0.146	1.90E-04
*HBS1L-MYB*	rs9376090	135411228	T>C	0	NA	NA	0.01	0.471	1.60E-02
*HBS1L-MYB*	rs9399137	135419018	T>C	0.04	0.412	0.0086	0.01	0.668	8.30E-06
*HBS1L-MYB*	rs9389269	135427159	T>C	0.18	0.09561	0.2468	0.03	0.4	1.40E-05
*HBS1L-MYB*	rs9402686	135427817	G>A	0.03	0.1447	0.4437	0.06	0.342	1.60E-04
*HBS1L-MYB*	rs9494142	135431640	T>C	0.11	0.3391	0.0023	0.13	0.085	6.00E-02
Chromosome 11									
*HBG2*	rs7482144	5276169	G>A	0	–0.05843	0.9076	0.01	0.562	1.60E-04
*OR51B5/6*	rs5006884	5373251	C>T	0.08	0.04163	0.7385	0.05	0.164	2.40E-02

NA, not applicable; monomorphic T for the entire sample; MAF, minor allele frequency; SNP, single-nucleotide polymorphisms. *Chromosome, position on NCBI Build 36.1.

Furthermore, studies in Cameroon and Tanzania lacked power to replicate the association of a sub-locus (rs7482144) in *HBG2* (Table 1), which explained 2.2% of the variation in HbF levels in African American patients.^8^ This is likely to be due to the absence of Senegal and Indian–Arab beta-globin locus haplotypes that contain the rs7482144 in most Cameroonian patients.^17^

Similarly, a strong signal adjacent to the *HBB* cluster recently detected in African-American patients at rs5006884 in *OR51B5/6^18^* was not found to have significant association in either Tanzanian^14^ or Cameroonian SCD patients.^13^ These findings suggest that studies of multiple SCD populations in Africa are warranted to improve our understanding of the impact of human diversity on HbF expression in SCD.^19^

## The co-inheritance of alpha-thalassaemia and SCD

The co-inheritance of α-thalassaemia is associated with a milder phenotype in patients with HbSS and Sβ^0^ thalassaemia, e.g. higher haemoglobin level and lower stroke rate.^20^ However, the effect of α-thalassaemia is not all positive; pain and aseptic necrosis may be higher.^21^

In Cameroon, the co-inheritance of α-thalassaemia and SCD was associated with late onset of clinical manifestations and potentially increased survival in Cameroonian patients; this could explain the much higher allele frequency of 3.7kb α-globin gene deletion among SCD patients than in controls.^22^,^23^ In Tanzania, the co-inheritance of α-thalassaemia and SCD was associated with a lower stroke risk.^24^

These preliminary data indicate an urgent need to replicate and expand genetic studies in many other African SCD populations, including studies focused on loci that are linked to stroke^25^ and other cardiovascular conditions, to fully measure the opportunities of their implementation to improve the care of patients with SCD.

## Addressing the burden of cardiovascular diseases in SCD in Africa

Cardiovascular phenotypes in SCD include complications involving the heart (e.g. heart failure), brain (e.g. stroke), lung (e.g. pulmonary hypertension) and kidney (e.g. proteinuria). Cerebrovascular disease is perhaps the most devastating complication for children with SCD, including overt stroke, transient ischaemic attacks, silent infarcts and neurocognitive dysfunction. Longitudinal cohort data from the USA have shown that between five and 10% of patients with SCD will experience a clinically overt stroke during childhood.26 The prevalence of overt stroke in SCD in Africa may be higher than that reported in high-income countries.

Overt stroke is a clinical diagnosis and should be easily detected in any cohort of closely monitored SCD patients. Brain computerised tomography (CT) and magnetic resonance imaging (MRI) are used to rule out haemorrhage or localise the tissue/vascular pathological basis for the stroke event. Clinical examination and CT scans identified a stroke prevalence of 6.7% in Cameroon.^27^,^28^ A study of children with SCD in Nigeria found a stroke prevalence of 8.7%.^29^

The prevalence of silent cerebral infarcts (SCI) and cerebral vasculopathies has been shown to be even greater than overt stroke risk: SCI occurs in 27% of this population before their sixth, and 37% by their 14th birthdays.^30^ SCI is diagnosed by MRI, but has not been studied in Africa because of the limited availability of MRI equipment. In fact SCI is not really silent, as falling school performance and other signs of neurocognitive dysfunction and change in personality/behaviour may all raise suspicion for increased risk of overt stroke, and suspicion of stroke with absence of motor or speech defect. SCI could be better called covert cerebral infarction.

The lack of longitudinally monitored SCD cohorts in Africa weakens incidence and prevalence estimates. Indeed, the cognitive performance of Cameroonian SCD children was evaluated using a neuropsychological test battery assessing four domains of cognitive functioning (executive function, attention, memory and sensory-motor skills). A high prevalence of cognitive deficits was found, increasing with age, and with a specific impairment of executive functions and attention.^31^ Up to 37.5% of the 96 SCD patients aged six to 24 years (M = 13.5, SD = 4.9) had mild-to-severe cognitive deficits, which tended to increase with age.

Structural equation models showed a significant association between (1) severe anaemia and lower executive functioning, (2) low foetal haemoglobin levels and lower executive functioning and attention, (3) history of cerebrovascular accidents and lower performances on executive functioning, sensory-motor and memory tasks, (4) pathological electroencephalogram and lower attention span, and (5) abnormal transcranial Doppler and lower memory function.^31^

The feasibility of using transcranial Doppler (TCD) ultrasonography in Africa to determine risk of stroke in children with SCD has been demonstrated in studies in Tanzania,^24^ Cameroon,^31^ Nigeria^32^ and Kenya.^33^ However, because of limited resources and inefficient transfusion services, TCD is seldom established as part of routine healthcare followed by transfusion therapy to prevent overt stroke in those found to have abnormal blood flow velocity.^33^

Pulmonary arterial hypertension (PAH) is common, with a prevalence of 30% in SCD patients, and all-cause mortality rates of 40% at 40 months after diagnosis in the USA.^34^ Studies in Nigeria indicate PAH could represent a significant complication of SCD on the African continent.^35^

N-terminal (NT) pro-brain natriuretic peptide (proBNP) ≥ 160 ng/l has a 78% positive predictive value for pulmonary hypertension. NT-proBNP elevation is common and is associated with markers of anaemia, inflammation and iron status and with severe functional impairment among sickle cell anaemia patients in Nigeria.^36^

The prevalence of elevated tricuspid regurgitant velocity (TRV) measured by echocardiogram, which predicts risk for pulmonary hypertension and death in adult sickle cell anaemia, was similar among SCD patients in Tanzania and those from the USA.^37^ In addition, there is accumulating clinical evidence to suspect a high prevalence of kidney disease among African SCD patients in France,^38^ Nigeria,^39^,^40^ Ghana^41^ and the Congo.^42^ The data revealed and emphasised the need to draft a specific research agenda to include Africa in future comprehensive studies on the epidemiology and genetics of end-organ complications of SCD.

## Addressing the genomics of cardiovascular diseases in SCD in Africa

Despite the evidence of a high burden of cardiovascular events in SCD patients, the magnitude of this problem in Africa has not been defined. The clinical variability and environmental factors influencing these events have not been clearly and systematically studied, despite the availability of some encouraging data on the genetics of these cardiovascular phenotypes of SCD among African populations from the diaspora (Table 2). Previous studies of sibling pairs have demonstrated a genetic component to the development of cerebrovascular disease in SCD stroke.^43^ In addition, a child with SCD had an increased risk for stroke if they had siblings who had experienced an overt stroke.^44^

**Table 2:** Selected genes associated with cardiovascular phenotypes among African American SCD patients

*Cardiovascular phenotypes in SCD*	*Associated genes*	*References*
Stroke	HBA (3.7 alphaglobin gene deletion)	Hsu et al. J Pediatr Hematol Oncol 2003; 25(8): 622–628
	GOLGB1 (Y1212C)	Flanagan et al. Blood 2013; 121(16): 3237–3245
	ENPP1 (K173Q)	
Kidney disease (proteinuria)	MYH9	Ashley-Koch et al. Br J Haematol 2011; 155(3): 386–394
	APOL1	
Pulmonary hypertension	GALNT13	Desai et al. Am J Respir Crit Care Med 2012; 186(4): 359–368
	ADORA2B	

A few genetic modifiers have confirmed the association with stroke, such as α-thalassaemia trait being protective against stroke^20^ (Table 1), but these do not explain the entire genetic contribution to stroke risk. In addition, several retrospective studies, mostly among African Americans, have identified specific SNPs associated with stroke in patients with SCD, using candidate gene approaches, but failed to be replicated using independent validation cohorts.^45^

Recent data that used genetic mapping and exome sequencing revealed that one mutation in *GOLGB1 (Y1212C)* and another mutation in *ENPP1* (K173Q) were confirmed as having significant associations with a decreased risk for stroke among African Americans with SCD^25^ (Table 1). These studies need to be validated and extended in SCD patients in Africa.

Like stroke, renal failure occurs in 5–18% of SCD patients and is associated with early mortality.^46^ At-risk SCD patients cannot be identified prior to the appearance of proteinuria. The myosin, heavy-chain 9, non-muscle (*MYH9*) and apolipoprotein L1 (*APOL1*) genes have been associated with risk for focal segmental glomerulosclerosis and end-stage renal disease in African Americans.^47^

Seven SNPs in MYH9 and one in APOL1 were significantly associated with proteinuria among African American SCD patients. In addition, glomerular filtration rate was negatively correlated with proteinuria (*p* < 0.0001), and was significantly predicted by an interaction between *MYH9* and *APOL1^48^* (Table 2). Further studies with independent data sets from sub-Saharan Africa are now needed to confirm this association, to identify more of the genes involved, and the interaction with various African environments, in order to address preventative measures of SCD nephropathy.

Moreover, an increased tricuspid regurgitation jet velocity (TRV > 2.5 m/s) and pulmonary hypertension defined by right heart catheterisation both independently conferred increased mortality in SCD.^34^ A preliminary genetic association study comparing patients with an elevated (*n* = 49) versus normal (*n* = 63) TRV revealed significant association with five SNPs within *GALNT13* (*p* < 0.005), and a quantitative trait locus upstream of the adenosine-A2B receptor gene (*ADORA2B*)^49^ (Table 2).

Limited genetic studies associated with these critical cardiovascular phenotypes in SCD (stroke, pulmonary hypertension, kidney disease) have not been reported in SCD patients who reside in Africa. This indicates an urgent need to perform these studies, which could inform the global SCD communities in a unique way, on the value of gene and environmental interactions in the pathogenesis and hopefully the care of SCD.

## Integrating outcomes of genetics research into new-born screening and interventions to reduce childhood mortality and survival in SCD

Major benefits in the health and survival of children with SCD have been attained through the implementation of a few simple, evidence-based interventions. The most striking achievements have resulted from early diagnosis of SCD through new-born screening and the subsequent enrolment of these patients into comprehensive care programmes. These programmes provide interventions that include prophylaxis against pneumococcal infection using penicillin, and early detection and treatment of acute clinical events such as anaemia, septicaemia, stroke and acute chest syndrome. These interventions have been introduced in a limited manner in Africa, despite the fact that they have been shown to be highly effective in developed countries.

Hydroxyurea, an important therapeutic intervention for SCD in high-income settings, is beginning to be used more frequently in several African countries.^50^-^53^ There is no doubt that hydroxyurea will have a large public health impact in Africa.^54^ However there are questions regarding the effectiveness of hydroxyurea in some individuals possessing characteristics associated with poor response to treatment. This includes SCD populations with low levels of haemolysis,^55^ low HbF level and Central African Republic (CAR) haplotype,^56^ as well as children under five years of age with SCD, even though some data indicate that efficacy is just as good or better in younger children.^57^ These questions should not delay the use of hydroxyurea in Africa, but it is strongly recommended that research trials should be conducted to monitor and evaluate effectiveness in this setting.

The second challenge regarding use of hydroxyurea in SCD in Africa is access due to limited supply and high cost. It has also been suggested that patients and families may resist adherence with this treatment. In Cameroon, only 3.4% of SCD patients had access to hydroxyurea.^58^ Sociological data on the barriers associated with prescription of and adherence with hydroxyurea is needed in order to plan effective strategies to address these issues in Africa.

Despite the limited access to hydroxyurea and other care and therapies, about 3% of the 700 studied Cameroonian patients with SCD lived longer than 40 years.^14^ Specific survivor SCD populations in sub-Saharan Africa can offer new research opportunities to uncover possible variation that could improve the life of SCD patients. With more and more genomic data available, it is anticipated that new-born screening could also allow early identification of genetic factors (e.g. HbF-promoting SNPs or stroke-associated SNPs) to potentially assess each individual patient’s risks and plan appropriate anticipatory guidance.

## Perspectives: H3Africa and opportunity for genomic research of cardiovascular diseases in SCD

Currently, H3Africa extends across African countries, comprising 23 grants. It is anticipated that, together, H3Africa projects will analyse samples from 50 000 to 75 000 participants. Specifically, three projects have the objective to study stroke, kidney disease and other cardiovascular diseases (rheumatic heart disease) in various African countries where SCD is also prevalent^59^ (e.g. Cameroon, Tanzania, Nigeria, Ghana, Mali, Uganda). These projects offer the opportunity to extend the existing network of researchers in Cameroon, Ghana, Nigeria, South Africa and Tanzania, which have been assembled to conduct multicentre, Africa-based studies on the genetics and genomics of SCD.

To strengthen the case for genomic studies in Africa, several genetic variations have been discovered through molecular studies on the African continent.^60^ There is enough evidence, including whole-genome data from African populations, that emphasises the high levels of genomic variation and the heterogeneity of African populations.^61^,^62^

Some of the tremendous genetic variation in Africa is responsible for problems in clinical management of SCD, such as red blood cell transfusion, red blood cell Rh D polymorphism and allo-immunisation,^63^ and response to medications (cytochrome P450 polymorphisms and codeine/other opioids for pain therapy).^64^ Polymorphisms in ribonucleotide reductase, the target enzyme for hydroxyurea, may have variable effects on SCD patient response and deserves further investigation in Africa.

One SCD project currently funded under the H3Africa umbrella is focused on research in Cameroon, Ghana and Tanzania (FOA: RM12-005, 1 U01 HG007459-01). The project aims to: (1) explore perspectives and attitudes regarding genomic research and its implementation and implications in Africa, and (2) assess perceptions about public health interventions to increase awareness, early detection and prevention of SCD-related complications. Beyond this project, the investigators are building on biological materials, preliminary clinical and genomics data from Cameroon, Tanzania, Nigeria and Ghana, and extending the experience to other African countries, with the goal to improve infrastructure for research and training. The ultimate goal is to conduct research to understand the relationship between genes, the environment and disease, in order to translate genome-based knowledge into health benefits for SCD patients and their families in Africa.

## Key messages

SCD is characterised by marked clinical variability, with genetic factors playing key modulating roles. Studies in Tanzania and Cameroon have reported that SNPs in the *BCL11A* loci and *HBS1L-MYB* region (*HMIP*), and co-inheritance of alpha-thalassaemia impact on HbF level and clinical severity.
There are several cardiovascular phenotypes in SCD, such as stroke, heart failure, pulmonary hypertension and renal disease that contribute to its morbidity and mortality.
The prevalence of overt stroke among SCD patients in Cameroon (6.7%) and Nigeria (8.7%) suggests a higher burden than in high-income countries.
The genetics of stroke, kidney disease and pulmonary hypertension have seldom been investigated in SCD in Africa.
Several H3Africa projects are focused on cardiovascular phenotypes, which creates a major opportunity to build on existing SCD work in Africa, a genome-based research on key cardiovascular phenotypes to transform the health benefits of SCD patients.
